# Supplementary material for: HOXA9 has the hallmarks of a biological switch with implications in blood cancers
Source: Nat Commun. 2022 Oct 3;13:5829. doi: 10.1038/s41467-022-33189-w (PMC9530117; doi:10.1038/s41467-022-33189-w)
Supplement: Supplementary file 1 — Supplementary Information [file 41467_2022_33189_MOESM1_ESM.pdf]

SUPPLEMENTARY APPENDIX

HOXA9 has the hallmarks of a biological switch with implications in blood cancers.

Talarmain et al.

Table of Contents

Supplemental Methods...2

Qualitative Networks...2

Computational network model construction: description and literature review...3

Gene network construction...3

Model genotype/phenotype specifications...5

RUNX1, MYB and MYC general expression trend in [36] are consistent with our model. 6

XGBoost...6

MPN mouse models...8

Lentiviral vectors and viral transfection...8

Haematopoietic stem cell (HSC) isolation...9

HOXA9 Gene knockdown...9

Colony-Forming Unit (CFU) Assays...10

Colony imaging and counting...10

Supplemental Figures...11

Supplemental Tables...27

Supplemental References...36

Supplemental Methods

Qualitative Networks

Qualitative networks are an extension of Boolean networks described in detail in [1]. Of note, the nodes representing molecular expression are discrete variables with more than two states. This means higher resolution in expression levels. Additionally, the activity of a variable is determined by an algebraic target function rather than a table of values. The formalism is defined below.

A Qualitative network $Q(C,T,N)$ consists of components in C which can take values in $\{0,1,..,N\}$. N is a constant integer with any possible values above one, and is called the node granularity. If N is one, the model is a Boolean network. T is the set of target functions, which are the functions that determines the level toward which each component moves at the following time step. At each time step, each component moves by a maximum of one level. The update of each component $c_i \in C$ can therefore be mathematically defined as:

$$c_i(t+1) = \begin{cases} c_i + 1 \text{ if } \mathrm{target_i}(s(t)) > c_i \\ c_i - 1 \text{ if } \mathrm{target_i}(s(t)) < c_i \\ c_i \quad\quad \text{ if } \mathrm{target_i}(s(t)) = c_i \end{cases}$$

with $s(t)$ the current state of the network and $\mathrm{target_i} \in T$ the target function of c_i. $\mathrm{target_i}$ returns a value in $\{0,1,..,N\}$. The default target function is the difference between the amount of activation $\mathrm{act_i}$ and the amount of inhibition $\mathrm{inh_i}$ a component c_i is subject to. Both are averaged by the total number of activating/inhibiting components and are defined as follows:

$$\mathrm{act_i} = \frac{\sum_{\alpha_{ji}>0} \alpha_{ji} c_j}{\sum_{\alpha_{ji}>0} \alpha_{ji}}$$

$$\mathrm{inh_i} = \frac{\sum_{\alpha_{ji}<0} \alpha_{ji} c_j}{\sum_{\alpha_{ji}<0} \alpha_{ji}}$$

All models are built using the BioModelAnalyzer (BMA) tool [2] (https://biomodelanalyzer.org/), and analysed using LTL [3] and stability analysis [4]. BMA includes two types of edges representing respectively the activation or the inhibition of a node.

Computational network model construction: description and literature review

To build and validate a computational network model, the process must iterate over three fundamental steps:

- Develop a "specification"- a set of specific biological phenotypes and gene activities associated with each input condition (for example, the genotype or environment)
- Create a network using genes of interest and their interactions as nodes and edges. Phenotypes are also modelled as nodes in the network
- Refine the "target functions" on each gene and phenotype that govern how the gene responds to the activity of upstream element

A "correct" model will match all input specifications. As the model is expanded to include extended specifications or more genes, this cycle of testing and refinement must be repeated. The MPN model includes four genotypes in the specifications; wild-type JAK2/TET2, TET2 loss of function, JAK2 gain of function, and the JAK2/TET2 double mutant (Table 1). Variables are discrete and range from zero to two, representing inactive, resting, and hyperactive states.

Gene network construction

Genes included in the network were selected based on their relationships with the three core genes (TET2, JAK2, and HOXA9), and their involvement in control of modelled phenotypes. *SPI1* and *CEBPA* have well defined roles in monocyte and macrophage lineages [5]. *SPI1*

activates *CEBPA* in early progenitors [6, 7] and *CEBPA* helps the transition from CMP to GMP [8]. Both genes are essential for myeloid differentiation and are downregulators of progenitor proliferation.

Differentiation is associated with a reduced cell proliferation [9]. Our model includes *MYB* and *E2F1* as upstream effectors for CMP and GMP expansion [10, 11] which are both inactivated by *SPI1* and *CEBPA* [12-14]. Furthermore, we include *GATA1* in our network as part of JAK2 pathway and its role in determining the erythroid lineage. Both GATA1 and JAK2 genes have been shown to be important players in MEP progenitor production and erythropoiesis [15, 16]. *GATA1* is additionally required for *KLF1* activation, a marker of erythroid differentiation [17]. KLF1 has also been established as a downstream target of phosphorylated *TET2* in erythroid cell lines and *TET2* phosphorylation is induced by *JAK2* [18]. *JAK2* is an upstream regulator of *GATA1* via AKT [19], but also plays an role in MEP expansion with *STAT5* and MAPK activation of the anti-apoptotic gene *BCL2L1* [20, 21].

Whilst *JAK2* is associated with erythroid differentiation, it also can play a role in determining the myeloid lineage through *STAT3* mediated activation of *SPI1* [22].

Finally, *SPI1* and *GATA1* mutually inhibit one another [23]. This feature is necessary for erythroid/myeloid lineage commitment [24]. We additionally include *RUNX1* as a link between those hematopoietic genes and our *JAK2*/*TET2*/HOXA9 motif through *RUNX1* activation by HOXA9. *RUNX1* upregulation has been associated with *HOXA9* upregulation in early stem and progenitor cells [25, 26] and therefore is a strong candidate to link our motif with our hematopoietic genetic regulators. *RUNX1* is found in the earliest stages of haematopoiesis which makes this gene essential for a fully functional haematopoiesis [27]. We therefore link

this gene to the rest of our network through its downstream targets *SPI1* for the myeloid lineage [28] and with *GATA1* for the erythroid lineage [29, 30].

<u>Model genotype/phenotype specifications</u>

The model specifications are defined in Table 1 in the paper and are described in more details in this section. In the wild-type state, the model is stable with all variables (genes and phenotypes) equal to one. In the single *TET2* loss-of-function mutation state, the cell has increased stem cell self-renewal [31], elevated CMP expansion [32] and diminished overall differentiation [33] with a skew towards the granulocyte-monocyte lineage [34]. This is tested in our model by looking for stability, with an increase in GMP expansion, whilst MEP expansion remains at its wild type levels. In the JAK2 mutant (overactivation) background, erythroid differentiation and MEP expansion are both increased [35, 36]. Furthermore, GMP expansion is also increased to reflect *JAK2* stimulation of myeloid cells, but GMP differentiation remains at wild-type levels as the erythroid lineage specifically is preferred [37].

Finally, the double mutant is a bifurcating system with two fixed points and no cyclic attractors. Each fixed point represents alternative orders of TET2/JAK2 mutations; that is to say, *TET2* first or *JAK2* first double mutants. We characterised those states using Ortmann et al [38]. Both fixed points have an increased stem cell self-renewal due to *TET2* loss. GMP and MEP expansion are also increased in both states as *TET2* loss promotes granulocyte-monocyte development, while *JAK2* favours the erythroid lineage. However, following observations in Ortmann et al [38], in our model *TET2* first mutants have an increased CMP expansion not observed in *JAK2* first. Additionally, the differentiation loss in *JAK2* single mutants is retrieved by *JAK2* overexpression, resulting in GMP differentiation being in its normal state in both double mutants. Experiments have shown that *JAK2* first double mutant patients have an increased number of mature erythroid cells, and so erythroid differentiation is increased in *JAK2* first but not *TET2* first double mutant. In total, both double mutants share four common

phenotypes. Only CMP expansion and erythroid differentiation differ between the two fixed points consistent with patient characteristics described in Ortmann et al [38].

RUNX1, MYB and MYC general expression trend in [36] are consistent with our model.

We plot *RUNX1, MYB* and *MYC* expression for the different genotypes in the data and compare them to their expression in our biological network. In Figure S1, S2 and S3, all genotypes come from [36] and therefore the genotypes "JAK2" and "TET2" refer to the single mutant mouse models, and "DM" is the double mutant with *JAK2* mutated first. "WT" designates the wild type (no mutation) genotype.

XGBoost

We use XGBoost (eXtreme Gradient Boosting) to rank different gene pathways according to their relationship to the expression of a gene of interest. We selected pathways that have well established roles in cancer, and seek to identify which specific pathways and component genes have the highest correlation with the gene of interest and its expression level in the AML patients (TCGA RNASeq) [39].

Following the selection of a specific gene of interest, we begin by splitting the patients into two groups of 30 patients with either highest or lowest expression of our gene. We use XGBoost binary classification to determine which pathways are the best to classify patients into the right cohort. Thirteen text files containing different subset of genes corresponding to popular cancer pathways were previously generated using the literature: cell cycle, EMT, MAPK, MYC, NFKB, NOTCH, NRF2, PI3K, RAS-RTK, TGF-β, TP53, WNT, and WNT downstream. Genes and papers used in this analysis are described in Table S4.

A model is then trained and validated for each pathway. We used the logarithmic loss (logloss) function as the standard metric for binary classification evaluation, and set the *colsample_bytree* parameter to 0.3. By setting this value to 0.3, we limit the subset of genes used at each iteration to a randomly selected subset of genes, corresponding to 30% of the

total. This can reduce the apparent accuracy of the classifier by hiding highly correlated genes, but also reduces the likelihood that the result of the classification is based on a small subset. As such, we aim to increase the robustness of the accuracy across the whole pathway.

We split the 173 AML patients into a training set (80%) and a validation set (20%). Each pathway is compared using their Matthew Correlation Coefficient (*MCC*). We choose the MCC score for its robustness property for groups of differing sizes. In our analyses, the MCC scores the competence of each pathway to classify our patients into the right cohort:

$$MCC = \frac{TP \times TN - FP \times FN}{TP + TN + FP + FN}$$

True Positive (TP) and True Negative (TN) are counts representing how many times a pathway has correctly classified a patient into respectively the high or low cohort. False Positive (FP) represents the number of times a pathway has classified a patient with low expression into the high cohort, and vice versa for the False Negative (FN). MCC score varies -1 and 1, but we translate it into percentage in our figures.

We further use SHAP (**SH**apley **A**dditive ex**P**lanations) to explain the output of our XGBoost models. SHAP aims to ease the interpretability of complex models by representing the importance of model features with shapley values [40]. Documentation on SHAP and how to use it can be found on github (http://github.com/slundberg/shap).

MPN mouse models

JAK2, TET2 and JAK2/TET2 double-mutant as published in Shepherd et al., Blood 2018 and wild-type (C57BL/6J, CD45.2) littermate controls mice were bred and maintained in the designated animal facilities of the Department of Biology at the University of York, UK. Mice were co-housed by sex in groups of 4–5 in individually ventilated cages on a 12:12 hour (7:00/19:00) light:dark cycle at 20–24°C with 45–65% humidity and provided water and food ad libitum. All experiments performed on mice were undertaken under UK Home Office Licence granted to Dr. Kent (PEAD116C1) which was approved by the local AWERB committee and UK Home Office.

Lentiviral vectors and viral transfection

Lentiviral vectors in a pGFP-C-shLenti backbone used in this study were a noneffective Scrambled shRNA control (TR30021, Origene) and four unique HOXA9 29mer shRNA constructs (TL500979-A, B, C and D). Viral production was performed with Lenti-X cells (HEK 293T, Takara Bio Inc), by co-transfection with a packaging plasmid pCMV-dR8.91 (Gag-Pol), a pCMV VSV-g envelope and the plasmid of interest, using the Fugene transfection reagent (Promega) diluted in OptiMEM media (Gibco). Cells were maintained in low glucose Dulbecco's Modified Eagle's Medium (DMEM, Gibco), supplemented with 10% FBS, 1% Penicillin/Streptomycin and 4 mM L-Glutamine (Gibco). Cells were incubated at 37°C, 5% CO2. Media was replaced at four hours and in the morning after transfection. Viral supernatant was harvested at 48 hours and 72 hours post-transfection. Supernatant was filtered through a 0.45-µm pore syringe filter. Cell-free supernatant was concentrated by addition of Lenti-X concentration solution (Takara Bio Inc), followed by centrifugation at 1500 g, 4°C for 45 minutes. Viral pellets were resuspended in Iscove's Modified Dulbecco's Medium (IMDM, Cytiva) and incubated for further 45 minutes at 4°C. Viral stocks were stored at -80°C until they were to be used.

Haematopoietic stem cell (HSC) isolation

Femurs, tibias and iliac crests from 3 wild-type, 3 JAK2, 3 TET2 and 3 JAK/TET mice were collected to prepare whole bone marrow suspensions. Red blood cell lysis was achieved by treatment with Ammonium Chloride solution (StemCell Technologies) and lineage depletion was performed by immunomagnetic selection of mature lineage cells using the haematopoietic stem progenitor cell (HSPC) enrichment cocktail and EasySep magnetic beads (StemCell technologies). Cell preparations were stained with lineage markers CD11b (Mac-1) and Ly6G/Ly6C (Gr-1) in PE-Cy7, Ly6-A/E (Sca-1) in BV421 and CD117 (c-Kit) in APC-Cy7 (Table S5, S6). All antibodies were purchased from Biolegend Inc. HSC-enriched fractions were obtained using the Lineage-negative, Sca-1 positive and c-Kit positive (LSK) compartment and were isolated by Fluorescence-Activated Cell Sorting (FACS – Fig. S14,S15) using the Moflo Astrios cell sorter (Beckman Coulter). Pools consisting of ~4000 LSK cells were sorted into individual wells of a 96-well plate containing an initial volume of 50 µL of StemSpan medium (StemCell Technologies) supplemented with 10% FBS, 1% Penicillin/Streptomycin, 1% L-glutamine (Gibco), 0.1 mM of 2β-Mercaptoethanol (Thermo Fisher), 300 ng/mL of Stem Cell Factor (SCF) and 20 ng/mL of interleukin (IL)-11 (StemCell Technologies).

HOXA9 Gene knockdown

Following isolation, LSK cells were incubated at 37°C, 5% CO2 for 30-60 minutes prior to transduction. For HOXA9 gene knockdown experiments, three biological replicates from each genotype for the two different conditions were used (noneffective scrambled control- and shHOXA9-transduced cells). Medium in each well was supplemented with Polybrene (Sigma Aldrich) at a final concentration of 10 ug/mL. Cells were transduced by adding cell-free viral supernatants and centrifuging the plates at 600 x g, for 30 minutes at 30°C. Cells were incubated overnight. The day after transduction, final volume in the wells was brought up to 200 µL of medium to dilute the polybrene reagent and viral stock. Cells were incubated for a total of 48 hours before isolation for in vitro functional assays.

Colony-Forming Unit (CFU) Assays

Plates containing transduced LSK cells were centrifuged for 5 minutes at 300 x g. Media was removed, and cells were rinsed with PBS (Sigma Aldrich) supplemented with 2% FBS (Gibco) by centrifugation as described above. CD45 in BV421 (Biolegend) was used to stain the cells prior to isolation by FACS. Live, CD45+, GFP+ LSK cells were sorted into Eppendorf tubes containing 300 µL of StemSpan medium (StemCell Technologies). The cell suspension was added into 2.7 mL of Methocult (methylcellulose) medium (StemCell Technologies), and cells were thoroughly vortexed for about 1 minute. The cell suspension was incubated for 10 minutes at room temperature. Following incubation, 1.5 mL of cell mixture in Methocult was added into each well of a 6-well SmartDish (StemCell Technologies) to have two duplicate wells from each genotype and each transduction condition. CFU assay plates were incubated at 37°C, 5% CO_2 for 14 days.

Colony imaging and counting

At day 14 of incubation, CFU assay plates were imaged on the StemVision system (StemCell Technologies). Colonies were characterised and counted after acquisition of high-quality images. Normalised number of colonies grown in each replicate was calculated per 100 colonies plated into each well. Statistical analysis to determine statistically significant differences was done through an unpaired Student’s t test (GraphPad Prism, v 9.0.2).

Supplemental Figures

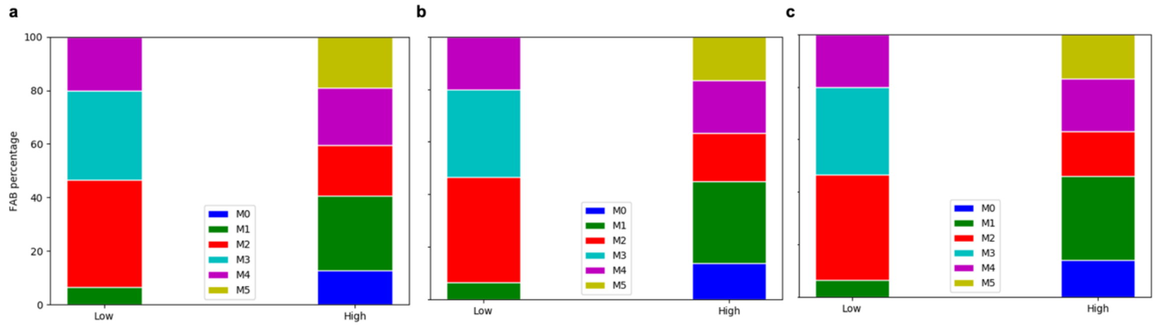

Figure S1. FAB distribution in *HOXA9* low and high patient cohorts for different high peak thresholds. The low-expressing cohort is kept constant at 0.005-1 log(TPM+1), whilst the high-expression cohort is defined by different boundaries. The threshold ranges, as expressed in log(TPM+1) : a) 4-5.5, b) 3.5-6 and c) 3-6.5. Those ranges were selected to represent the high peak and to be around the mean value of the peak located at about 4.5 log(TPM+1).

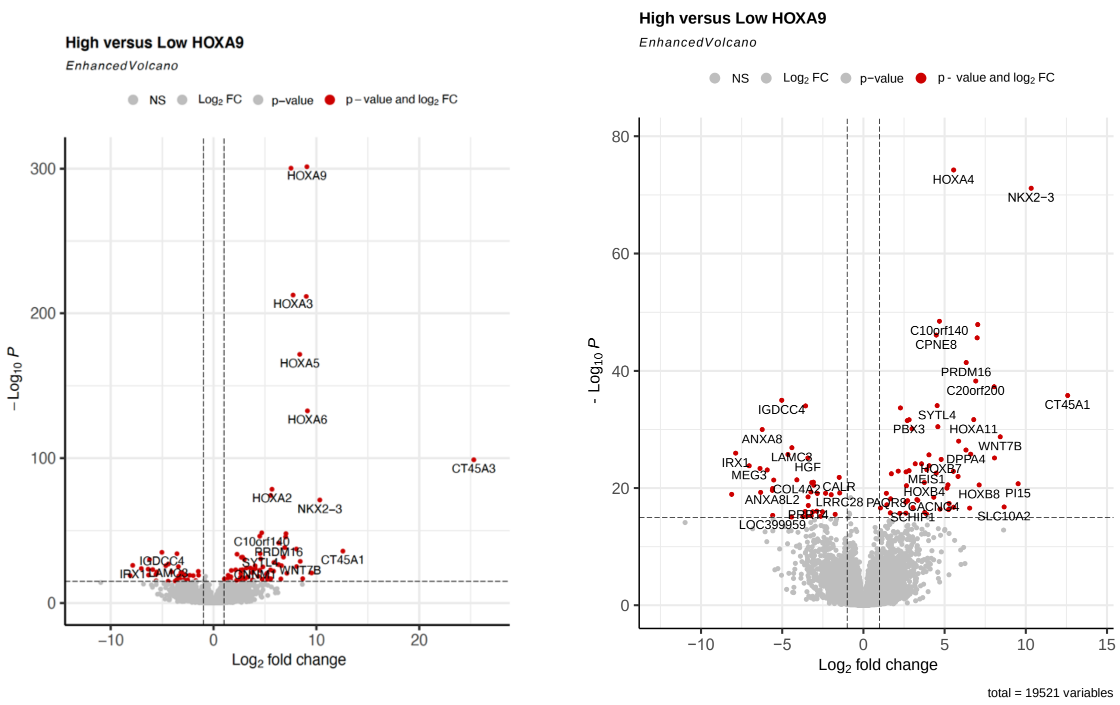

Figure S2. Differential gene expression analyses do not show specific hematopoietic function difference between HOXA9 cohorts. Genes with high log2 fold-change are found upregulated in the high HOXA9 peak. As expected, the HOX family is showing high fold changes between cohorts. Right figure is the zoomed-in volcano plot.

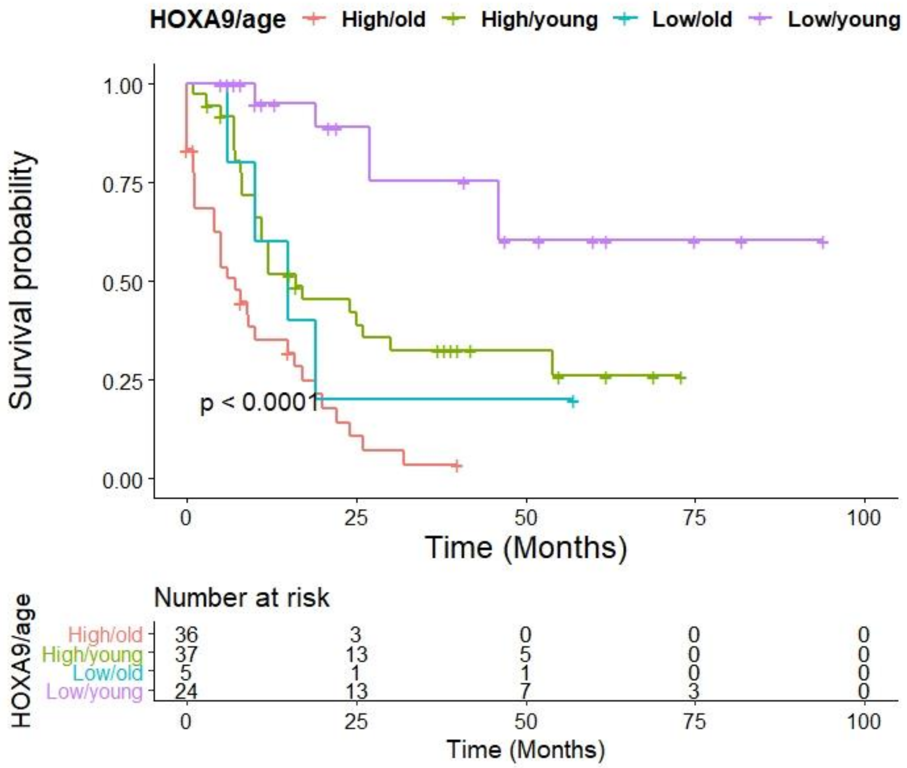

Figure S3. ***HOXA9*** **high and low cohorts have divergent AML prognosis regardless of age.** We use log-rank test to compare all groups. Survival is significantly different among groups ($p = 3.9 \times 10^{-8}$). We find that young patients (below 60 years old) with high HOXA9 expression have a poorer survival probability (22 months) compared to young patients with low expression (26 months – $p = 0.005$).

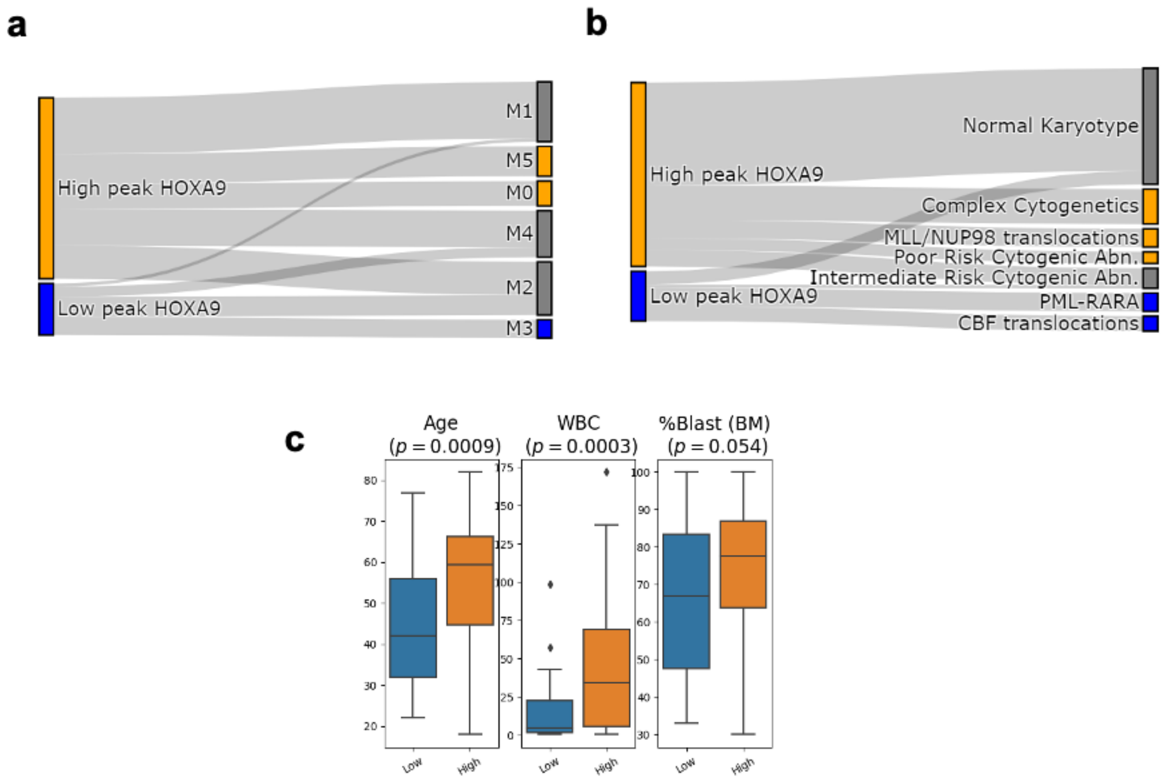

Figure S4. ***HOXA9* low and high expression can be used to stratify patients with AML.** HOXA9 expression partially explains (a) French-American-British (FAB) and (b) molecular classifications of 121 AML patients. Using Sankey diagrams, we find that the M3 FAB subtype as well as the PML-RARα and CBF (CBFB-MYH11 and RUNX1-RUNXT1) translocations are solely linked to low expression of HOXA9 (31 patients). Similarly, the high cohort (80 patients) possesses specific AML subclasses: M0, M5, MLL/NUP98 translocations and complex cytogenetics. FAB classification clusters AML patients into 8 groups from M0 the most undifferentiated subtype, M3 and M5 the subtypes with the higher number of monocytic/granulocytic blasts and M6-M7 (not included here due to the low number of patients) associated to the erythroid and megakaryocyte lineages. (c) HOXA9 cohorts show distinct clinical characteristics: high cohort patients (80) are older, display a higher white blood cell counts (WBC) and tend to have higher percentage of blasts in the bone marrow (two-sided Wilcoxon-Mann-Whitney statistical test). Box plots represent interquartile range and whiskers 1.5 * IQR.

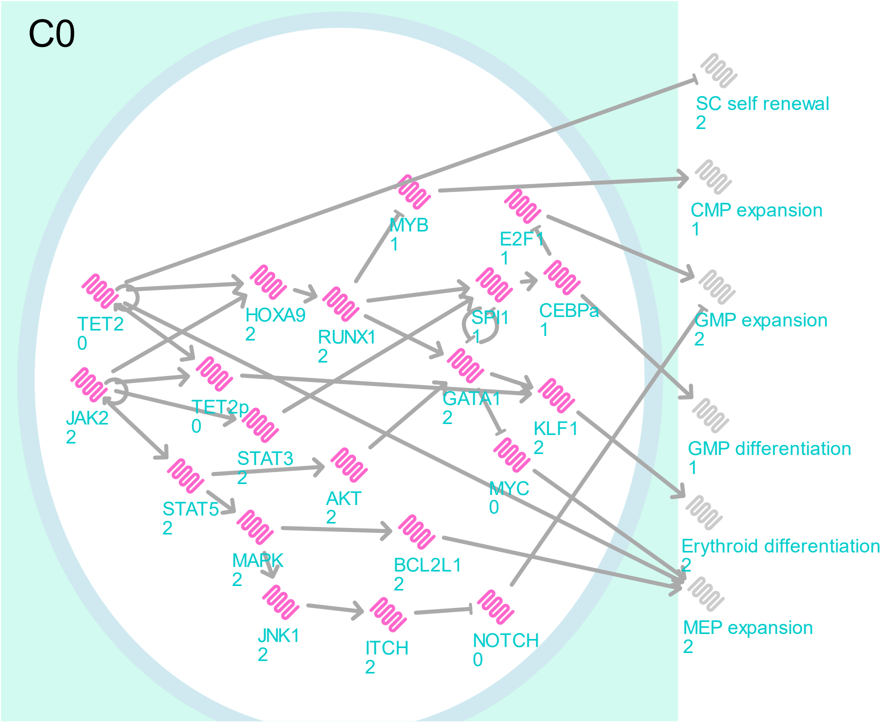

Figure S5. Loss of *HOXA9* feedback loop results in its overexpression and loss of bifurcation in the double mutant. In this model, when both *JAK2* and *TET2* are mutated, the phenotype is similar to the JAK2-first patients, where the number of differentiated cells increases which is not observed in patients with a first *TET2* mutation [38].

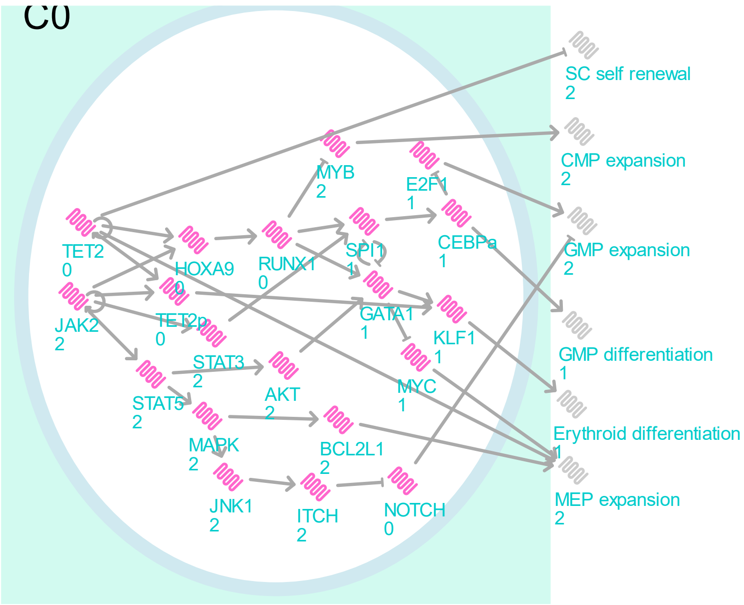

Figure S6. Loss of *HOXA9* positive feedback loop can result in *HOXA9* null activity. In this model, when both *JAK2* and *TET2* are mutated, the phenotype is similar to the TET2-first patients, where the number of common myeloid progenitors (CMP) increases which is not observed in patients with a first *JAK2* mutation [38].

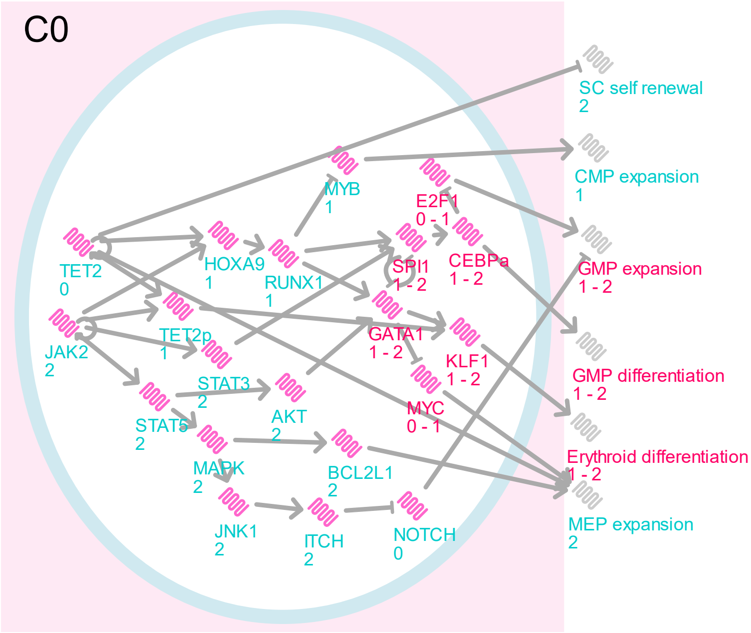

Figure S7. Loss of *HOXA9* positive feedback loop results in its wild-type expression while restoring the bifurcation in the double mutant, and introducing cycles. In this scenario, CMP expansion is stable which is an observed clinical characteristic of patients with different mutation orders for *JAK2* and *TET2* mutations [38].

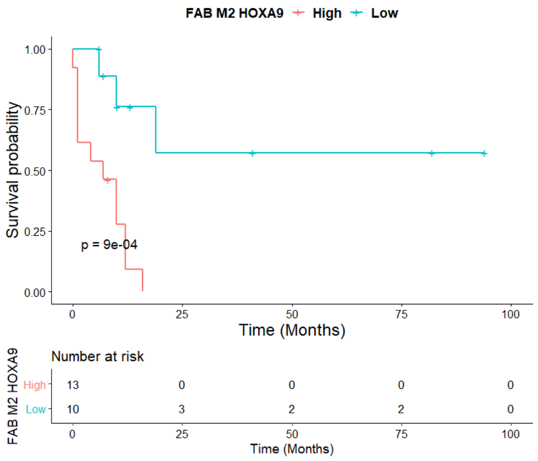

Figure S8. *HOXA9* high and low cohorts of M2 AML patients show distinct survival probabilities. None of the 13 patients with high expression for *HOXA9* survive past 20 months while three among the 10 patients with low expression reach 25 months (log-rank test - $p = 0.0009$).

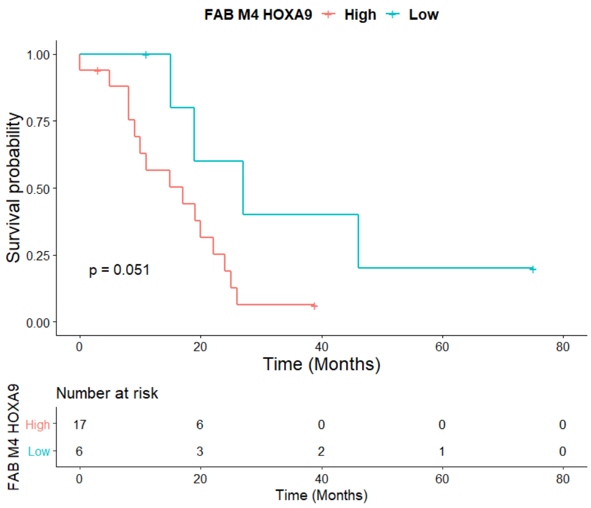

Figure S9. *HOXA9* high and low cohorts of M4 AML patients show distinct survival probabilities. The Kaplan Meier curves for the survival of M4 patients indicates a trend towards lower survival probability for patients with high *HOXA9* expression compared to the low-*HOXA9* cohort (log-rank test - $p = 0.051$).

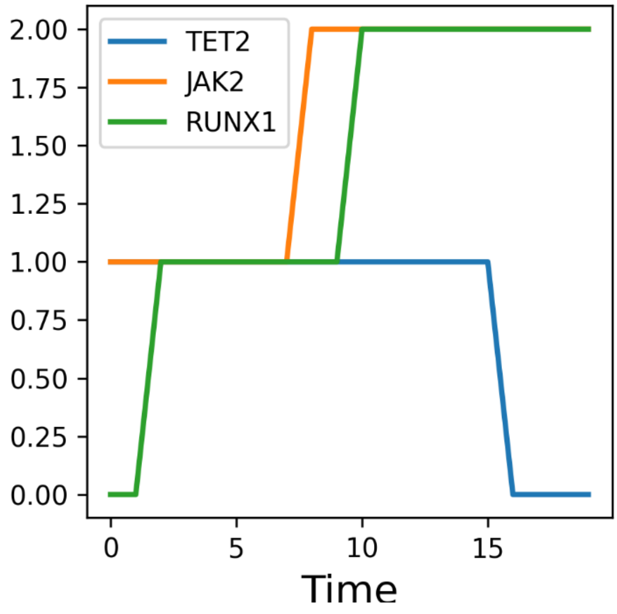

Figure S10. *RUNX1* expression in JAK2 first patients. *JAK2* activation mutation increases *RUNX1* expression when *TET2* is unmutated.

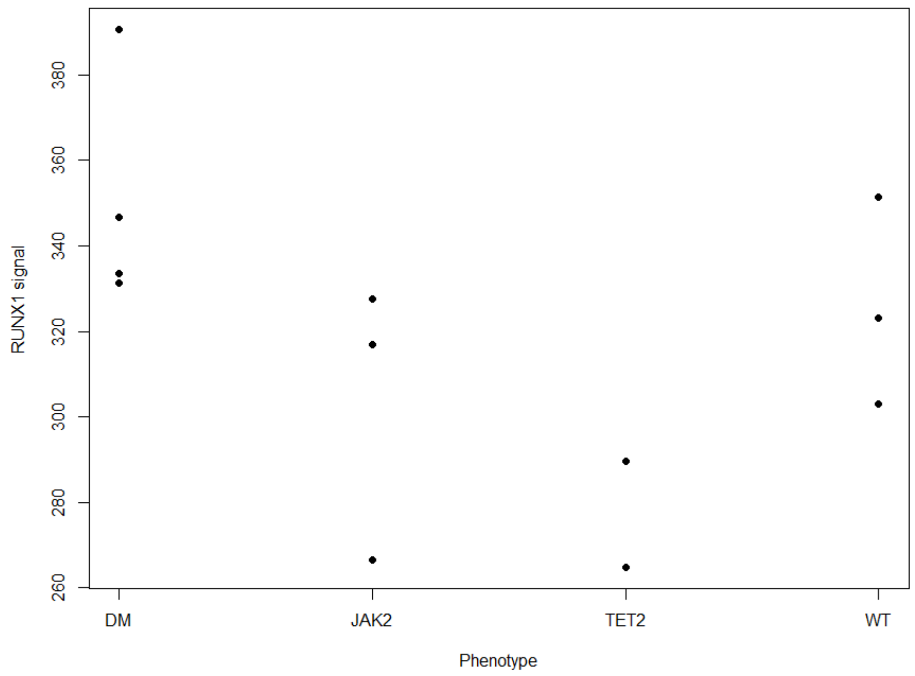

Figure S11. *RUNX1* expression in public MPN data follow the same trend as our model. Our model predicts *RUNX1* to be higher in the *JAK2* single and *JAK2* first double mutants and lower in *TET2* single mutants compared to the wild type (Table S2). Despite the low number of data points, the trends for *RUNX1* expression in the different genotypes are consistent with our findings.

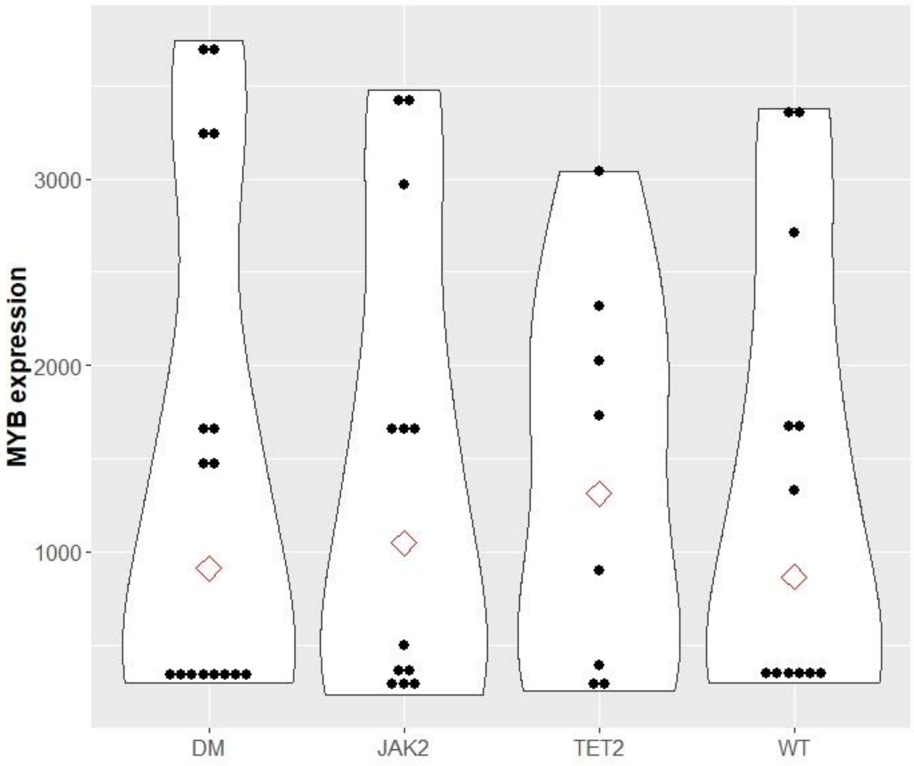

Figure S12. *MYB* expression in public MPN data follow the same trend as our model. Our model predicts *MYB* expression to be higher in the *TET2* single mutants while *JAK2* single and *JAK2* first double mutants have a similar *MYB* expression compared to the wild type genotype (Table S2). Despite the low number of data points, using the violin figure we identify that the trends for *MYB* expression in the different genotypes are consistent with our findings. Red rectangles represent the median expression.

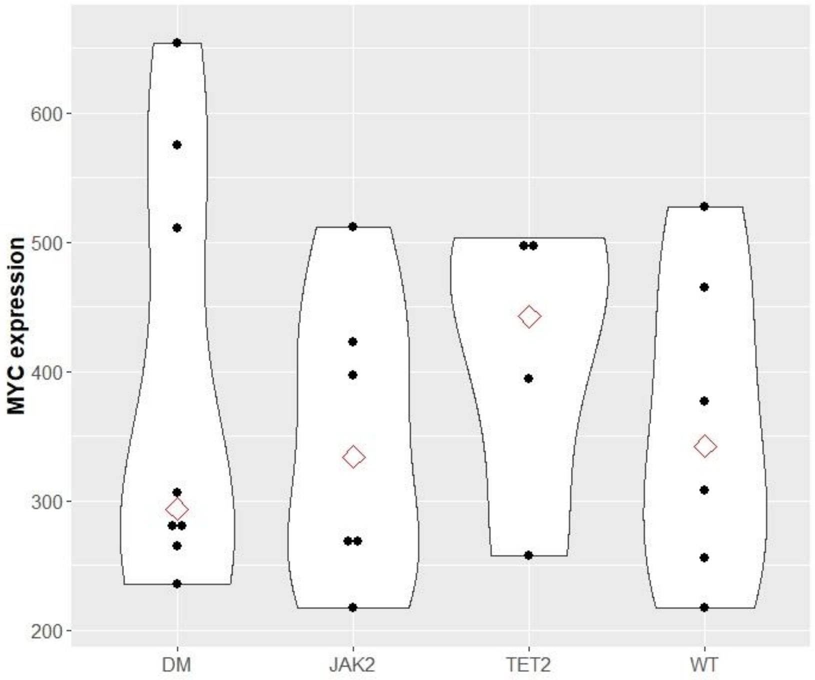

Figure S13. ***MYC*** **expression in public MPN data follow the same trend as our model.** Our model predicts *MYC* expression to be higher in the *TET2* single mutants while *JAK2* single and *JAK2* first double mutants should show a lower expression compared the wild type genotype (Table S2). Despite the low number of data points, using the violin figure we identify that the trends for *MYC* expression in the different genotypes fit our findings for the *TET2* single mutant and the *JAK2* first double mutant. Red rectangles represent the median expression.

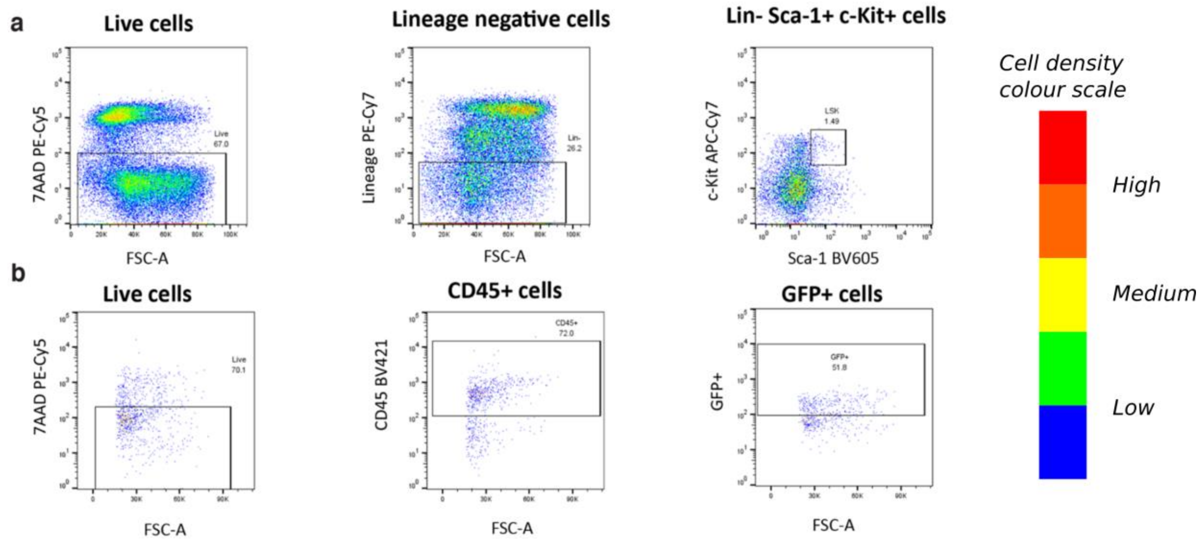

Figure S14. Fluorescence activated cell sorting (FACS) isolation strategies for: (a) Lineage-negative, Sca-1+ and c-Kit+ (LSK) cells and (b) CD45+ GFP+ LSK cells. Populations are gated from live cells, and show the LSK and CD45+ and GFP+ gates. Cells were stained using the antibody panels and concentrations described in the text and Table S5,S6.

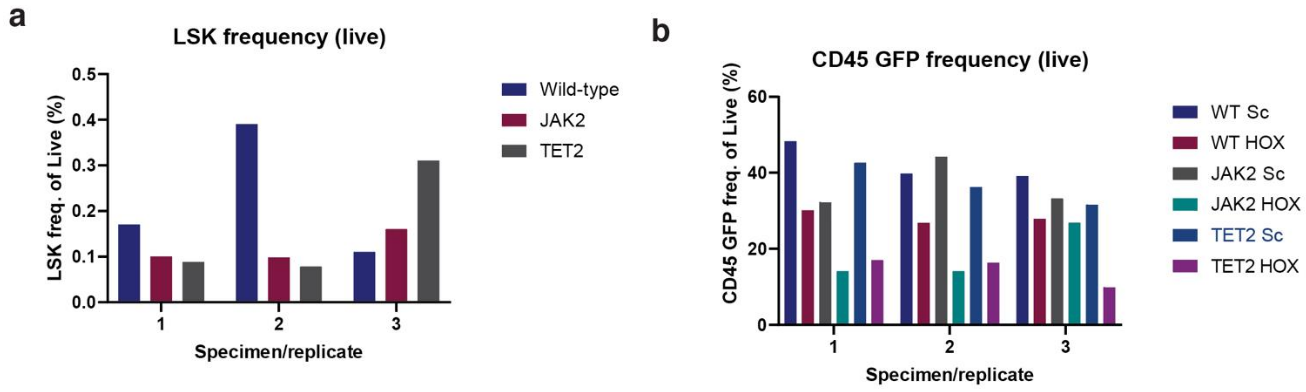

Figure S15. Cell population abundance in all samples and both conditions, expressed as % of live cells: (a) Lineage-negative, Sca-1+ and c-Kit+ (LSK) cells and (b) CD45+ GFP+ LSK cells. Sc: Scrambled.

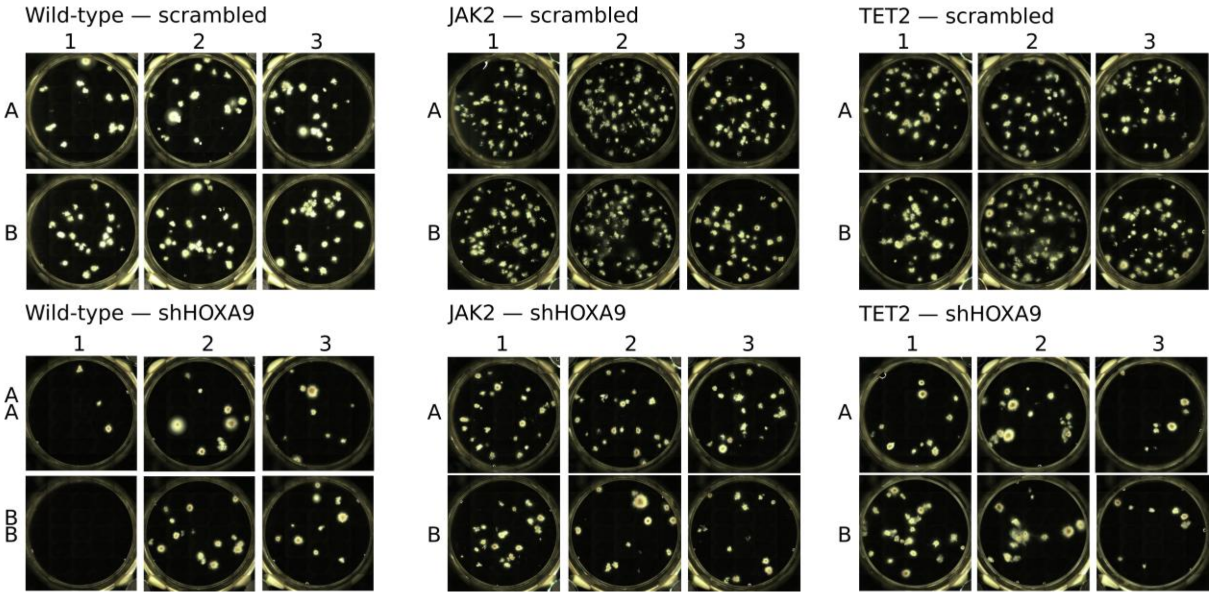

Figure S16. Colony-forming unit assay images: Each condition has three biological replicates (1,2,3) and two technical replicates (A,B).

Supplemental Tables

**Upstream gene**	**Interaction type**	**Downstream gene**	**Reference**
TET2	Activates	*HOXA9*	[41]
JAK2	Activates	*HOXA9*	[42]
HOXA9	Activates	*HOXA9*	[43]
HOXA9	Activates	*RUNX1*	[25, 26]
RUNX1	Activates	*SPI1*	[28]
RUNX1	Activates	*GATA1*	[29, 30]
SPI1	Inhibits	*GATA1*	[23]
GATA1	Inhibits	*SPI1*	[23]
SPI1	Activates	*CEBPa*	[6]
CEBPa	Inhibits	*E2F1*	[13]
CEBPa	Activates	GMP differentiation	[8]
E2F1	Activates	GMP expansion	[11]
RUNX1	Inhibits	*MYB*	Model prediction
MYB	Activates	CMP expansion	[10]
TET2	Activates	SC self-renewal	[31]
GATA1	Activates	*KLF1*	[17]
GATA1	Inhibits	*MYC*	[44]
MYC	Activates	MEP expansion	[44]
KLF1	Activates	Erythroid differentiation	[17]
TET2	Activates	*TET2p*	[18]
JAK2	Activates	*TET2p*	[18]
TET2p	Activates	*KLF1*	[18]
JAK2	Activates	*STAT3*	[45]
JAK2	Activates	*STAT5*	[45]

STAT3	Activates	*SPI1*	[22]
STAT5	Activates	*AKT*	[19]
AKT	Activates	*GATA1*	[19]
STAT5	Activates	*MAPK*	[20]
MAPK	Activates	*BCL2L1*	[20]
BCL2L1	Activates	MEP expansion	[21]
TET2	Activates	MEP expansion	[34]
MAPK	Activates	*JNK1*	[46]
JNK1	Activates	*ITCH*	[47]
ITCH	Inhibits	*NOTCH*	[48]
NOTCH	Inhibits	GMP expansion	[49]

Table S1. Gene interaction table for *JAK2*/*TET2* BMA model. Studies are derived from human and mouse experiments. All interactions are derived from studies of blood cells.

	JAK2 mutant	TET2 mutant	JAK2 first DM	TET2 first DM
JAK2	2	1	2	2
TET2	1	0	0	0
TET2p	2	0	0	0
HOXA9	2	0	2	0
RUNX1	2	0	2	0
SPI1	1	0	1	1
GATA1	2	0	2	1
MYB	1	2	1	2
E2F1	1	2	1	1
CEBPa	1	0	1	1
KLF1	2	0	2	1
MYC	0	2	0	1
STAT3	2	1	2	2
STAT5	2	1	2	2
AKT	2	1	2	2
MAPK	2	1	2	2
JNK1	2	1	2	2
ITCH	2	1	2	2
NOTCH	0	1	0	0
BCL2L1	2	1	2	2

Table S2. Gene values in the single and double mutant end states. In the unmutated state, all variables have the value 1.

Node	Target Function	References	Comments
HOXA9	JAK2*TET2 + 2*max(0,(HOXA9-1))*max(0,JAK2-1)	[42],[41],[43]	Activation by *JAK2*, *TET2* and itself. Memory property prediction.
SPI1	STAT3 + min((RUNX1-1) , (1-GATA1))	[28], [23],[19]	Activation by *STAT3* and *RUNX1* and inhibition loop with *GATA1*. Minimum function to have normal *SPI1* expression when *GATA1* is underexpressed but decreased expression when *GATA1* is overexpressed independently of *RUNX1*.
GATA1	AKT+RUNX1-max(SPI1,1)	[29, 23],[22]	Activation by *AKT* and *RUNX1* and inhibition loop with *SPI1*. Maximum function necessary as loss of *SPI1* does not increase erythroid differentiation.
MEP expansion	min(BCL2L1+TET2 , max(MYC, BCL2L1))	[36],[44],[21],[34],[35]	*TET2* loss reduces erythroid progenitors by skewing toward myeloid lineage. *JAK2* positive regulation of the erythroid lineage is stronger than *TET2* through *MYC* activation.

Table S3. Target functions of the model variables. Except for the nodes indicated by this table, all nodes have the default target function of the BioModelAnalyzer (BMA) tool which is the difference between the average state of all the variables activating the current node and the

average of all the variables inhibiting it. If there is no activating variable in the model, the target function equals the difference between a constant representing the basal activity of the node and the inhibiting variables. The constant is calculated so that in the healthy state (no mutation), all variables equal 1.

Pathways	References	Genes
Cell Cycle	[50]	*‘CDKN1A’, ‘CDKN1B’, ‘CDKN2A’, ‘CDKN2C’, ‘CCND2’, ‘CCND3’, ‘CCNE1’, ‘CDK2’, ‘CDK4’, ‘CDK6’, ‘RB1’, ‘E2F1’, ‘E2F3’*
EMT	[51]	*‘VIM’, ‘SNAI1’, ‘FN1’, ‘MMP2’*
MAPK	[52]	*‘GRB2’, ‘SOS1’, 'SOS2', 'HRAS', 'KRAS', 'BRAF', 'RAF1', 'MAP2K1', 'MAP2K2', 'MAPK3', 'MAPK1'*
MYC	[50]	*'MAX', 'MGA', 'MLX', 'MLXIP', 'MNT', 'MXD1', 'MXD3', 'MXD4', 'MXI1', 'MYC', 'MYCN'*
NFKB	[53]	*'NFKB1', 'NFKB2', 'RELA', 'RELB', 'REL', 'IKBKB', 'IKBKE', 'IKBKG'*
NOTCH	[50]	*'ARRDC1', 'CREBBP', 'EP300', 'KAT2B', 'KDM5A', 'NOTCH1', 'NOTCH2', 'NOTCH4', 'NRARP', 'PSEN2', 'LFNG', 'ITCH', 'NCSTN', 'SPEN', 'JAG1', 'APH1A', 'FBXW7', 'FHL1', 'HDAC2', 'CUL1', 'RFNG', 'NCOR1', 'NCOR2', 'HDAC1', 'NUMB', 'MAML3', 'MFNG', 'CIR1', 'MAML1', 'MAML2', 'NUMBL', 'PSEN1', 'PSENEN', 'RBPJ', 'RBX1', 'SAP30', 'SKP1', 'SNW1', 'CTBP1', 'CTBP2', 'ADAM10', 'APH1B', 'ADAM17', 'DTX2', 'DTX3L', 'DTX4', 'EGFL7'*
NFRF2	[50]	*'NFE2L2', 'KEAP1', 'CUL3'*
PI3K	[50]	*'EIF4EBP1', 'AKT1', 'AKT2', 'AKT3', 'AKT1S1', 'DEPDC5', 'INPP4B', 'MAPKAP1', 'MLST8', 'MTOR', 'NPRL2', 'NPRL3', 'PDK1', 'PIK3CA', 'PIK3CB', 'PIK3R1', 'PIK3R2', 'PPP2R1A', 'PTEN', 'RHEB', 'RICTOR', 'RPTOR', 'RPS6', 'RPS6KB1', 'STK11', 'TSC1', 'TSC2'*
RTK-RAS	[50]	*'SPRED2', 'SHOC2', 'PPP1CA', 'SCRIB', 'PIN1', 'KSR1', 'PEBP1', 'ERF', 'PEA15', 'JAK2', 'IRS2'*

TGF-Beta	[50]	*'TGFBR1', 'TGFBR2', 'ACVR1B', 'SMAD2', 'SMAD3', 'SMAD4'*
TP53	[50]	*'TP53', 'MDM2', 'MDM4', 'ATM', 'CHEK2', 'RPS6KA3'*
WNT	[50]	*'CHD8', 'LEF1', 'LRP5', 'LZTR1', 'PORCN', 'TLE1', 'TLE3', 'TLE4', 'CTNNB1', 'DVL1', 'DVL2', 'DVL3', 'FRAT1', 'FRAT2', 'FZD1', 'FZD2', 'FZD5', 'FZD6', 'APC', 'AXIN1', 'GSK3B', 'RNF43', 'TCF7', 'TCF7L2', 'CHD4'*
WNT downstream	[54]	*'VIM', 'APOE', 'LILRB1', 'LEF1', 'ARHGAP4', 'MAP4K2', 'TCF4', 'BIRC5', 'CDC25B', 'MSL1', 'TCF7', 'PEPD', 'CDKN2A', 'MYC', 'BTRC', 'PROCR', 'MYCN', 'ENPP2', 'TGIF1', 'RUNX2', 'MITF', 'DUSP6', 'LBH', 'FN1', 'PDCL', 'LMO2', 'MYCBP', 'PLCG2', 'EFNB1', 'RHOU', 'MMP2', 'ID2', 'RARG', 'VCAN', 'IFI30', 'IL1B', 'VEGFA', 'IL32', 'UBE2I', 'CD44', 'ITGB7', 'TSHR', 'ENC1', 'JUN', 'PTTG1', 'CREM', 'SNAI1', 'SIAH2', 'PLAUR', 'CYBB', 'BGLAP', 'PPARD', 'FOSL1', 'DUSP1', 'FOS', 'PTGS2', 'JAG1', 'KLF10'*

Table S4. Selected pathways and genes used in the XGBoost analyses.

Antibody	Fluorochrome	Clone	Cat. Number	Concentration	Volume/sample (100 ul)
**Sca-1**	BV421	D7	BioLegend 108127	1:100	**1**
**c-Kit**	APC-Cy7	2B8	BioLegend 105826	1:100	**1**
**CD11b**	PE-Cy7	M1/70	BioLegend 101216	1:200	**0.5**
**Gr-1**	PE-Cy7	RB6-8C5	BioLegend 108416	1:200	**0.5**
**PBS**	---	---	---	---	**94 ul**
**7AAD**	----	---	Invitrogen A1310	1:1000	*1:1000*
				Total volume/mouse	**100 ul**

Table S5. Antibody panel for LSK isolation

Antibody	Fluorochrome	Clone	Cat. Number	Concentration	Volume/sample (50 ul)
**CD45**	BV421	30-F11	BioLegend 103133	1:100	**0.5**
**PBS**	---	---	---	---	**49.5 ul**
**7AAD**	----	---	Invitrogen A1310	1:1000	*1:1000*
				Total volume/mouse	**50 ul**

Table S6. Antibody panel for LSK isolation

Supplemental References

[1] M. A. Schaub, T. A. Henzinger, and J. Fisher, “Qualitative networks: a symbolic approach to analyze biological signaling networks,” *BMC systems biology*, vol. 1, no. 1, p. 4, 2007.

[2] D. Benque, S. Bourton, C. Cockerton, B. Cook, J. Fisher, S. Ishtiaq, N. Piterman, A. Taylor, and M. Y. Vardi, “Bma: Visual tool for modeling and analyzing biological networks,” in *International Conference on Computer Aided Verification*. Springer, 2012, pp. 686–692.

[3] A. Pnueli, “The temporal logic of programs,” in *18th Annual Symposium on Foundations of Computer Science (sfcs 1977)*. IEEE, 1977, pp. 46–57.

[4] B. Cook, J. Fisher, E. Krepska, and N. Piterman, “Proving stabilization of biological systems,” in *International Workshop on Verification, Model Checking, and Abstract Interpretation*. Springer, 2011, pp. 134–149.

[5] F. Rosenbauer and D. G. Tenen, “Transcription factors in myeloid development: balancing differentiation with transformation,” *Nature Reviews Immunology*, vol. 7, no. 2, pp. 105–117, 2007.

[6] P. Burda, N. Curik, J. Kokavec, P. Basova, D. Mikulenkova, A. I. Skoultchi, J. Zavadil, and T. Stopka, “Pu. 1 activation relieves gata-1–mediated repression of cebpa and cbfb during leukemia differentiation,” *Molecular Cancer Research*, vol. 7, no. 10, pp. 1693–1703, 2009.

[7] S. Pundhir, F. K. B. Lauridsen, M. B. Schuster, J. S. Jakobsen, Y. Ge, E. M. Schoof, N. Rapin, J. Waage, M. S. Hasemann, and B. T. Porse, “Enhancer and transcription factor dynamics during myeloid differentiation reveal an early differentiation block in cebpa null progenitors,” *Cell reports*, vol. 23, no. 9, pp. 2744–2757, 2018.

[8] P. Zhang, J. Iwasaki-Arai, H. Iwasaki, M. L. Fenyus, T. Dayaram, B. M. Owens, H. Shigematsu, E. Levantini, C. S. Huettner, J. A. Lekstrom-Himes *et al.*, “Enhancement of hematopoietic stem cell repopulating capacity and self-renewal in the absence of the transcription factor c/ebpα,” *Immunity*, vol. 21, no. 6, pp. 853–863, 2004.

[9] A. D. Friedman, “Runx1, c-myb, and c/ebpα couple differentiation to proliferation or growth arrest during hematopoiesis,” *Journal of cellular biochemistry*, vol. 86, no. 4, pp. 624–629, 2002.

[10] Y. K. Lieu and E. P. Reddy, “Impaired adult myeloid progenitor cmp and gmp cell function in conditional c-myb-knockout mice,” *Cell Cycle*, vol. 11, no. 18, pp. 3504–3512, 2012.

[11] B. T. Porse, D. Bryder, K. Theilgaard-Monch, M. S. Hasemann, K. Anderson, I. Damgaard, S. E. W. Jacobsen, and C. Nerlov, “Loss of c/ebpα cell cycle control increases myeloid progenitor proliferation and transforms the neutrophil granulocyte lineage,” *The Journal of experimental medicine*, vol. 202, no. 1, pp. 85–96, 2005.

[12] T. Bellon, D. Perrotti, and B. Calabretta, “Granulocytic differentiation of normal hematopoietic precursor cells induced by transcription factor pu. 1 correlates with negative regulation of the c-myb promoter,” *Blood, The Journal of the American Society of Hematology*, vol. 90, no. 5, pp. 1828–1839, 1997.

[13] B. T. Porse, T. Å. Pedersen, X. Xu, B. Lindberg, U. M. Wewer, L. Friis-Hansen, and C. Nerlov, “E2f repression by c/ebpα is required for adipogenesis and granulopoiesis in vivo,” *Cell*, vol. 107, no. 2, pp. 247–258, 2001.

[14] L. M. Johansen, A. Iwama, T. A. Lodie, K. Sasaki, D. W. Felsher, T. R. Golub, and D. G. Tenen, “c-myc is a critical target for c/ebpα in granulopoiesis,” *Molecular and cellular biology*, vol. 21, no. 11, pp. 3789–3806, 2001.

[15] X. Han, J. Zhang, Y. Peng, M. Peng, X. Chen, H. Chen, J. Song, X. Hu, M. Ye, J. Li *et al.*, “Unexpected role for p19ink4d in posttranscriptional regulation of gata1 and modulation of human terminal erythropoiesis,” *Blood, The Journal of the American Society of Hematology*, vol. 129, no. 2, pp. 226–237, 2017.

[16] H. Neubauer, A. Cumano, M. Müller, H. Wu, U. Huffstadt, and K. Pfeffer, “Jak2 deficiency defines an essentialdevelopmental checkpoint in definitivehematopoiesis,” *Cell*, vol. 93, no. 3, pp. 397–409, 1998.

[17] F. Lohmann and J. J. Bieker, “Activation of eklf expression during hematopoiesis by gata2 and smad5 prior to erythroid commitment,” *Development*, vol. 135, no. 12, pp. 2071–2082, 2008.

[18] J. J. Jeong, X. Gu, J. Nie, S. Sundaravel, H. Liu, W.-L. Kuo, T. D. Bhagat, K. Pradhan, J. Cao, S. Nischal *et al.*, “Cytokine-regulated phosphorylation and activation of tet2 by jak2 in hematopoiesis,” *Cancer discovery*, vol. 9, no. 6, pp. 778–795, 2019.

[19] I. Geron, A. E. Abrahamsson, C. F. Barroga, E. Kavalerchik, J. Gotlib, J. D. Hood, J. Durocher, C. C. Mak, G. Noronha, R. M. Soll *et al.*, “Selective inhibition of jak2-driven erythroid differentiation of polycythemia vera progenitors,” *Cancer cell*, vol. 13, no. 4, pp. 321–330, 2008.

[20] M. Socolovsky, A. E. Fallon, C. Brugnara, and H. F. Lodish, “Fetal anemia and apoptosis of red cell progenitors in stat5a-/- 5b-/- mice: a direct role for stat5 in bcl-xl induction,” *Cell*, vol. 98, no. 2, pp. 181–191, 1999.

[21] M. Mori, M. Uchida, T. Watanabe, K. Kirito, K. Hatake, K. Ozawa, and N. Komatsu, “Activation of extracellular signal-regulated kinases erk1 and erk2 induces bcl-xl up-regulation via inhibition of caspase activities in erythropoietin signaling,” *Journal of cellular physiology*, vol. 195, no. 2, pp. 290–297, 2003.

[22] A. D. Panopoulos, D. Bartos, L. Zhang, and S. S. Watowich, “Control of myeloid-specific integrin αmβ2 (cd11b/cd18) expression by cytokines is regulated by stat3-dependent activation of pu. 1,” *Journal of Biological Chemistry*, vol. 277, no. 21, pp. 19001–19007, 2002.

[23] P. Zhang, G. Behre, J. Pan, A. Iwama, N. Wara-Aswapati, H. S. Radomska, P. E. Auron, D. G. Tenen, and Z. Sun, “Negative cross-talk between hematopoietic regulators: Gata proteins repress pu. 1,” *Proceedings of the National Academy of Sciences*, vol. 96, no. 15, pp. 8705–8710, 1999.

[24] P. Burda, P. Laslo, and T. Stopka, “The role of pu. 1 and gata-1 transcription factors during normal and leukemogenic hematopoiesis,” *Leukemia*, vol. 24, no. 7, pp. 1249–1257, 2010.

[25] S. Tsuzuki and M. Seto, “Expansion of functionally defined mouse hematopoietic stem and progenitor cells by a short isoform of runx1/aml1,” *Blood, The Journal of the American Society of Hematology*, vol. 119, no. 3, pp. 727–735, 2012.

[26] V. Azcoitia, M. Aracil, C. Martnez-A, and M. Torres, “The homeodomain protein meis1 is essential for definitive hematopoiesis and vascular patterning in the mouse embryo,” *Developmental biology*, vol. 280, no. 2, pp. 307–320, 2005.

[27] J. D. Growney, H. Shigematsu, Z. Li, B. H. Lee, J. Adelsperger, R. Rowan, D. P. Curley, J. L. Kutok, K. Akashi, I. R. Williams *et al.*, “Loss of runx1 perturbs adult hematopoiesis and is associated with a myeloproliferative phenotype,” *Blood*, vol. 106, no. 2, pp. 494–504, 2005.

[28] Y. Huang, K. Sitwala, J. Bronstein, D. Sanders, M. Dandekar, C. Collins, G. Robertson, J. MacDonald, T. Cezard, M. Bilenky *et al.*, “Identification and characterization of hoxa9 binding sites in hematopoietic cells,” *Blood, The Journal of the American Society of Hematology*, vol. 119, no. 2, pp. 388–398, 2012.

[29] K. E. Elagib, F. K. Racke, M. Mogass, R. Khetawat, L. L. Delehanty, and A. N. Goldfarb, “Runx1 and gata-1 coexpression and cooperation in megakaryocytic differentiation,” *Blood*, vol. 101, no. 11, pp. 4333–4341, 2003.

[30] T. Yokomizo, K. Hasegawa, H. Ishitobi, M. Osato, M. Ema, Y. Ito, M. Yamamoto, and S. Takahashi, “Runx1 is involved in primitive erythropoiesis in the mouse,” *Blood, The Journal of the American Society of Hematology*, vol. 111, no. 8, pp. 4075–4080, 2008.

[31] L. Cimmino, I. Dolgalev, Y. Wang, A. Yoshimi, G. H. Martin, J. Wang, V. Ng, B. Xia, M. T. Witkowski, M. Mitchell-Flack *et al.*, “Restoration of tet2 function blocks aberrant self-renewal and leukemia progression,” *Cell*, vol. 170, no. 6, pp. 1079–1095, 2017.

[32] H. Kunimoto, Y. Fukuchi, M. Sakurai, K. Sadahira, Y. Ikeda, S. Okamoto, and H. Nakajima, “Tet2 disruption leads to enhanced self-renewal and altered differentiation of fetal liver hematopoietic stem cells,” *Scientific reports*, vol. 2, p. 273, 2012.

[33] M. Ko, H. S. Bandukwala, J. An, E. D. Lamperti, E. C. Thompson, R. Hastie, A. Tsangaratou, K. Rajewsky, S. B. Koralov, and A. Rao, “Ten-eleven-translocation 2 (tet2) negatively regulates homeostasis and differentiation of hematopoietic stem cells in mice,” *Proceedings of the National Academy of Sciences*, vol. 108, no. 35, pp. 14566–14571, 2011.

[34] K. Moran-Crusio, L. Reavie, A. Shih, O. Abdel-Wahab, D. Ndiaye-Lobry, C. Lobry, M. E. Figueroa, A. Vasanthakumar, J. Patel, X. Zhao *et al.*, “Tet2 loss leads to increased hematopoietic stem cell self-renewal and myeloid transformation,” *Cancer cell*, vol. 20, no. 1, pp. 11–24, 2011.

[35] C. H. Jamieson, J. Gotlib, J. A. Durocher, M. P. Chao, M. R. Mariappan, M. Lay, C. Jones, J. L. Zehnder, S. L. Lilleberg, and I. L. Weissman, “The jak2 v617f mutation occurs in hematopoietic stem cells in polycythemia vera and predisposes toward erythroid differentiation,” *Proceedings of the National Academy of Sciences*, vol. 103, no. 16, pp. 6224–6229, 2006.

[36] E. Chen, R. K. Schneider, L. J. Breyfogle, E. A. Rosen, L. Poveromo, S. Elf, A. Ko, K. Brumme, R. Levine, B. L. Ebert *et al.*, “Distinct effects of concomitant jak2v617f expression and tet2 loss in mice promote disease progression in myeloproliferative neoplasms,” *Blood, The Journal of the American Society of Hematology*, vol. 125, no. 2, pp. 327–335, 2015.

[37] H. Akada, D. Yan, H. Zou, S. Fiering, R. E. Hutchison, and M. G. Mohi, “Conditional expression of heterozygous or homozygous jak2v617f from its endogenous promoter

induces a polycythemia vera–like disease," *Blood, The Journal of the American Society of Hematology*, vol. 115, no. 17, pp. 3589–3597, 2010.

[38] C. A. Ortmann, D. G. Kent, J. Nangalia, Y. Silber, D. C. Wedge, J. Grinfeld, E. J. Baxter, C. E. Massie, E. Papaemmanuil, S. Menon *et al.*, "Effect of mutation order on myeloproliferative neoplasms," *New England Journal of Medicine*, vol. 372, no. 7, pp. 601–612, 2015.

[39] T. Chen and C. Guestrin, "Xgboost: A scalable tree boosting system," in *Proceedings of the 22nd acm sigkdd international conference on knowledge discovery and data mining*, 2016, pp. 785–794.

[40] S. M. Lundberg and S.-I. Lee, "A unified approach to interpreting model predictions," in *Advances in neural information processing systems*, 2017, pp. 4765–4774.

[41] M. T. Bocker, F. Tuorto, G. Raddatz, T. Musch, F.-C. Yang, M. Xu, F. Lyko, and A. Breiling, "Hydroxylation of 5-methylcytosine by tet2 maintains the active state of the mammalian hoxa cluster," *Nature communications*, vol. 3, no. 1, pp. 1–12, 2012.

[42] L. Bei, C. Shah, H. Wang, W. Huang, L. C. Platanias, and E. A. Eklund, "Regulation of cdx4 gene transcription by hoxa9, hoxa10, the mll-ell oncogene and shp2 during leukemogenesis," *Oncogenesis*, vol. 3, no. 12, pp. e135–e135, 2014.

[43] X. Zhong, A. Prinz, J. Steger, M.-P. Garcia-Cuellar, M. Radsak, A. Bentaher, and R. K. Slany, "Hoxa9 transforms murine myeloid cells by a feedback loop driving expression of key oncogenes and cell cycle control genes," *Blood advances*, vol. 2, no. 22, pp. 3137–3148, 2018.

[44] V. Munugalavadla, L. C. Dore, B. L. Tan, L. Hong, M. Vishnu, M. J. Weiss, and R. Kapur, "Repression of c-kit and its downstream substrates by gata-1 inhibits cell proliferation during erythroid maturation," *Molecular and cellular biology*, vol. 25, no. 15, pp. 6747–6759, 2005.

[45] W. J. Leonard and J. J. O'Shea, "Jaks and stats: biological implications," *Annual review of immunology*, vol. 16, no. 1, pp. 293–322, 1998.

[46] H.-L. Huang, M.-J. Hsieh, M.-H. Chien, H.-Y. Chen, S.-F. Yang, and P.-C. Hsiao, "Glabridin mediate caspases activation and induces apoptosis through jnk1/2 and p38 mapk pathway in human promyelocytic leukemia cells," *PLoS One*, vol. 9, no. 6, p. e98943, 2014.

[47] E. Gallagher, M. Gao, Y.-C. Liu, and M. Karin, "Activation of the e3 ubiquitin ligase itch through a phosphorylation-induced conformational change," *Proceedings of the National Academy of Sciences*, vol. 103, no. 6, pp. 1717–1722, 2006.

[48] P. Chastagner, A. Israel, and C. Brou, "Aip4/itch regulates notch receptor degradation in the absence of ligand," *PloS one*, vol. 3, no. 7, 2008.

[49] A. Klinakis, C. Lobry, O. Abdel-Wahab, P. Oh, H. Haeno, S. Buonamici, I. van De Walle, S. Cathelin, T. Trimarchi, E. Araldi *et al.*, "A novel tumour-suppressor function for the notch pathway in myeloid leukaemia," *Nature*, vol. 473, no. 7346, pp. 230–233, 2011.

[50] F. Sanchez-Vega, M. Mina, J. Armenia, W. K. Chatila, A. Luna, K. C. La, S. Dimitriadoy, D. L. Liu, H. S. Kantheti, S. Saghafinia *et al.*, "Oncogenic signaling pathways in the cancer genome atlas," *Cell*, vol. 173, no. 2, pp. 321–337, 2018.

[51] Y. Chae, D. Kim, S. W. Kim, and Y.-J. An, “Trophic transfer and individual impact of nano-sized polystyrene in a four-species freshwater food chain,” *Scientific reports*, vol. 8, no. 1, pp. 1–11, 2018.

[52] D. K. Morrison, “Map kinase pathways,” *Cold Spring Harbor perspectives in biology*, vol. 4, no. 11, p. a011254, 2012.

[53] P. N. Moynagh, “The nf-κb pathway,” *Journal of cell science*, vol. 118, no. 20, pp. 4589–4592, 2005.

[54] A. Koval and V. L. Katanaev, “Dramatic dysbalancing of the wnt pathway in breast cancers,” *Scientific reports*, vol. 8, no. 1, pp. 1–10, 2018.
